# Solitary fibrous tumor of the male breast: a case report and review of the literature

**DOI:** 10.1186/1477-7819-6-16

**Published:** 2008-02-07

**Authors:** Francesca Rovera, Giovanna Imbriglio, Giorgio Limonta, Marina Marelli, Stefano La Rosa, Fausto Sessa, Gianlorenzo Dionigi, Luigi Boni, Renzo Dionigi

**Affiliations:** 1 Department of Surgical Sciences, Ospedale di Circolo, Varese, Italy; 2 Department of Pathology, Ospedale di Circolo, Varese, Italy; 3 Department of Human Morphology, University of Insubria Varese and Department of Pathology, Multimedica, Milano, Italy

## Abstract

Extrapleural solitary fibrous tumors are very rare and occasionally they appear in extraserosal soft tissues or parenchymatous organs. In such cases the right preoperative diagnosis is often difficult and challenging, because both radiological and cytological examinations are not exhaustive. For these reasons, surgical excision is frequently the only way to reach the correct diagnosis and to achieve definitive treatment. A few cases of solitary fibrous tumors have been also described in the breast. Although rare, this lesion opens difficulties in preoperative diagnosis entering in differential diagnosis with other benign lesions as well as with breast cancer. In this article we describe a case of a solitary fibrous tumor of the breast in a 49-year-old man. Problems related to differential diagnosis and the possible pitfalls that can be encountered in the diagnostic iter of such rare tumor are discussed.

## Case presentation

**Figure 1:**
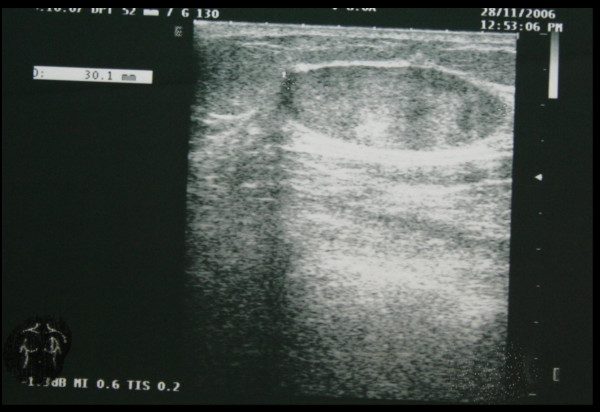
Breast ultrasound showed in the right retroareolar region, a solid mass of 3 × 1 cm with homogeneous echostructure and well-defined margins.

**Figure 2:**
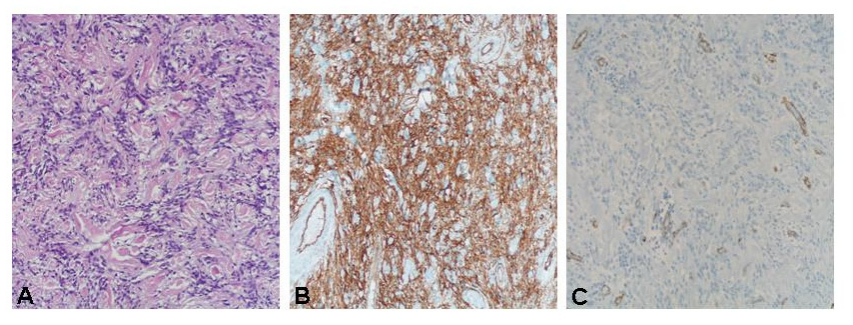
**A,B,C: **The tumor consists of a proliferation of bland-looking cells admixed with thin collagen fibers. Cell appearance ranged from fibroblastic-like cells with elongated nuclei and scanty cytoplasm (A). Cells were immunoreactive for CD34 (B), while they were completely negative for smooth muscle actin (C).

A 49-year-old white man presented at Department of Surgical Sciences of the University of Insubria in January 2007 due to a palpable painless nodule of the right breast, that he occasionally detected 3 months before. The patient had a positive family history for breast cancer (his mother was affected at the age of 55 years). His personal and pathological anamnesis did not highlight any significant evidence. Physical examination showed a lump of about 3 cm in the retroareolar region of the right breast, with well-defined margins, tense elastic consistence on palpation, mobile without skin or nipple-areola complex alterations. No ipsilateral axillary nodes have been detected. Breast ultrasound and fine-needle aspiration were performed. Breast ultrasound showed in the right retroareolar region, a solid mass of 3 × 1 cm with homogeneous echostructure and well-defined margins (fig. 1). These clinical and radiological data were highly suggestive for fibroadenoma. In cytological specimens only benign duct cells were observed. A surgical treatment was planned, with both diagnostic and therapeutic goals. The patient underwent surgical resection of the lesion in March 2007. Macroscopically, tumor presented as a white-grayish well demarcated unencapsulated nodule of 28 mm in diameter. Histologically, the lesion was composed of a proliferation of bland-looking cells admixed with thin collagen fibers. Cell appearance ranged from fibroblastic-like cells with elongated nuclei and scanty cytoplasm, to epitheliod-like oval cells with abundant eosinophilic cytoplasm and round to oval, centrally located, nuclei. No mitoses were found as well as areas of necrosis or hemorrhage. Immunohistochemical stains, performed using the avidin-biotin complex procedures, showed immunoreactivity for vimentin and CD34, while cells were completely negative for S100-protein, α-smooth muscle actin, desmin, cytoheratin AE1/AE3, and neurofilaments (fig. 2 A,B,C). On the basis of these morphological and immunohistochemical findings the diagnosis of solitary fibrous tumor was made.

## Discussion

Fibrous tumors involving the mammary gland are uncommon and account for less than 0.2% of all primary breast lesions, without a striking difference of incidence between male and female as for ductal epithelial cancers [1]. The majority of cases described in the literature occurred in the thoracic cavity, but various sites, including head and neck [2], liver [3], skin [4], soft tissue [5,6] and meninges [7,8], were recognized. Extraserosal solitary fibrous tumor can be included in the group of benign spindle stromal tumors of the breast, which encompasses a spectrum of lesions sharing several basic common clinical, morphological, and immunohistochemical analogies [9]. Tumors with similar features have been reported in the literature with different names, frequently used interchangeably, creating confusion of terminology among pathologists and clinicians. The unifying morphological criterion of all these lesions is represented by a well-circumscribed proliferation of bland-looking spindly to oval-epithelioid cells forming short fascicles and/or clusters, admixed with thick or thin collagen bands. Recently, Magro et al. proposed to subdivide these tumors in two main groups: the fibroblastic and myofibroblastic types (2002). Although both categories have a basic common immunophenotype characterized by immunoreactivity for vimentin, CD34, Bcl2 and CD99, they differentiate for the expression of myogenic markers including α-smooth muscle actin and desmin, lacking in the former and strongly expressed in the latter one [9,10]. Main morphological features of mesenchymal lesions of the breast are described in table 1.

**Table 1:** Main morphological features of mesenchymal lesions of the breast

Tumor type	atypia	vascular component	hemorrhage	necrosis	mitoses	CK	EMA	Vim	CD34	Bcl2	CD99	actin	desmin	S100
Solitary fibrous tumor	no	prominent	no	no	rare	-/+	+/-	+	+	+	+	-/+	-/+	-/+
Myofibroblastoma	no	present	no	no	rare	-	-	+	+/-	+/-	+/-	+	+	-
Fibromatosis	no	scarce	no	no	rare	-		+				-/+	-	-
Hemangiopericytoma	mild	abundant	no	rare	variable			+	+/-		+/-	-/+	-/+	
Nodular fascitis	no	abundant	red cell extravasion	no	present	-						+	+/-	-
Inflammatory myofibroblastic tumor *	mild	abundant	no	no		-/+		+				+/-	+	-
Leiomyoma	no	normal	no	no	rare	-	-	+	-	-	-	+	+	-
Metaplastic carcinoma	yes	normal	no	rare	present	+	-/+	+	-			+		-
Myoepithelioma	mild	normal	no	no	present	+			-			+	-	+
Pseudoangiomatous stromal hyperplasia	no	pseudovascular spaces	no	no	no	-	-	+	+	-	-	+	-	-

CK: cytokeratin; EMA: epithelial membrane antigen; Vim: vimentin; *: ALK positive

The interest of the present case relies on its rarity and in the difficulties to achieve the exact diagnosis, because this tumor has no typical radiological features and cytological aspects cannot frequently solve the diagnostic doubts between benign and malignant lesion. Tumors appear as single nodules, generally with well defined borders and enter in differential diagnosis with other more common lesions, including fibroadenomas and fillodes tumors. Moreover, breast cancer cannot be ruled out on the basis of radiological features. Cytology can help in the differential diagnosis from breast cancer, but could not in differentiating from other mixed epithelial-mesenchymal tumors. For all these reasons, the exact diagnosis is frequently achieved after surgical resection, that also has a curative purpose. Histologically, differential diagnosis of solitary fibrous tumors includes a wide variety of other benign and malignant bland-looking monomorphic spindle cell lesions of the breast, including nodular fascitis, inflammatory myofibroblastic tumor, fibromatosis, benign peripheral nerve sheet tumors, haemangiopericytomas and leiomyomas [10-12]. The differential diagnosis includes breast myofibroblastoma that shows the same morphological features, but differentiates for the expression of muscle-related antigens such as actin and desmin [9,10]. For this reason immunohistochemistry is a main tool in order to reach a correct diagnosis. However, the two entities have substantially the same clinical and biological behavior. Furthermore, differential diagnosis of a breast mass in a male routinely must distinguish from gynecomastia, which remains the most common cause of either unilateral or bilateral breast mass, frequently associated to hormonal therapy. Although more commonly bilateral and symmetric with well-defined discoid margins, histopatologic confirmation is the only sure differential between benign and malignant disease.

The differential diagnosis from cancer is the most important issue due to the very different prognostic implication. Although in surgical specimen this differential diagnosis is generally easy, on small bioptic or cytological specimens it may be difficult. In particular the detection in such preparations of epithelioid cells arranged in Indian files may mimic an infiltrating lobular carcinoma. Immunohistochemistry showing negativity for epithelial markers helps in excluding the presence of a breast cancer.

The treatment of choice for solitary fibrous tumours is extensive surgical resection. Up to now there is no evidence that chemotherapy and radiation are effective. The local recurrence or onset of metastases mainly depends on histological parameters. Although most solitary fibrous tumours are characterized by a non-aggressive clinical course, some can recur locally or display malignant behaviour, so a strict and long-term follow-up is recommended mainly for atypical forms.
